# The effect of smoking on bariatric surgical 30-day outcomes: propensity-score-matched analysis of the MBSAQIP

**DOI:** 10.1007/s00464-020-07838-4

**Published:** 2020-07-29

**Authors:** Michał R. Janik, Amir H. Aryaie

**Affiliations:** 1 Bariatric Center of Excellence, Department of Surgery, Texas Tech University Health Science Center, Lubbock, TX USA; 2 Department of General, Oncologic, Metabolic and Thoracic Surgery, Military Institute of Medicine, Szaserów 128, 04-141 Warszawa, Poland; 3 Bariatric and Reflux Center, Georgia SurgiCare, Atlanta, GA USA

**Keywords:** Bariatric surgery, Laparoscopic sleeve gastrectomy, Laparoscopic Roux-y gastric bypass, Metabolic and bariatric surgery accreditation, Quality improvement program (MBSAQIP), Smoking

## Abstract

**Background:**

The exact impact of smoking within the last 12 months on the safety outcome of sleeve gastrectomy and Roux-Y gastric bypass is not well known. The study aimed to assess the effects of smoking on 30-day surgical outcomes.

**Methods:**

Preoperative characteristics and outcomes from the Metabolic and Bariatric Surgery Accreditation and Quality Improvement Program dataset 2015–2018 were selected for all patients who underwent primary sleeve gastrectomy or Roux-Y gastric bypass. 30-day postoperative outcomes were assessed. We used propensity score matching to control for potential confounding.

**Results:**

In laparoscopic sleeve gastrectomy group, 29 165 pairs were included in the analysis. Smoking increased risk for inpatients readmission rate (3.67% vs. 3.10%; RR, 1.18; 95%CI 1.08–1.29, *p* < 0.001), intervention rate (1.03% vs. 0.84%; RR, 1.22; 95%CI 1.00–1.24, *p* = 0.020), reoperation rate (0.99% vs. 0.79%; RR, 1.25; 95%CI 1.05–1.48, *p* = 0.010), and leak rate (0.59% vs. 0.32%; RR, 1.83; 95%CI 1.43–2.37, *p* < 0.001). In laparoscopic Roux-Y gastric bypass cohort,11 895 pairs were included in the ultimate analysis. Smoking increased risk for inpatients readmission rate (7.54% vs. 5.88%; RR, 1.28; 95%CI 1.16–1.41, *p* < 0.001), intervention rate (3.53% vs. 2.30%; RR, 1.54; 95%CI 1.32–1.80, *p* < 001), reoperation rate (3.17% vs. 1.86%; RR, 1.70; 95%CI 1.45–2.00, *p* < 0.001), leak rate (1.05% vs. 0.59%; RR, 1.78; 95%CI 1.33–2.39, *p* < 0.001), bleed rate (2.03% vs. 1.45%; RR, 1.39; 95%CI 1.15–1.69, *p* < 0.001), and morbidity (4.20% vs. 3.38%; RR, 1.24; 95%CI 1.09–1.41, *p* = 0.001).

**Conclusion:**

Smoking cigarettes at any point within the 12 months before admission for surgery increased the risk for surgical short-term complications in bariatric patients. The effect was the most significant regarding leaks.

Smoking status is a modifiable behavior, and smoking cessation affects outcomes significantly in surgical patients [1]. Many surgeons require to stop smoking before surgery and refuse to operate smokers. However, active smoking is not a contraindication for bariatric surgery. Some patients undergo surgery as active smokers. A link between smoking status and increased complication rate was described in previous studies [2, 3]. However, the effect of smoking within the last 12 months on the safety outcome of the most popular bariatric procedures—Laparoscopic Sleeve Gastrectomy (LSG) and Laparoscopic Roux-Y Gastric Bypass (LRYGB) had not been estimated yet.

We utilized data from the Metabolic and Bariatric Surgery Accreditation and Quality Improvement Program (MBSAQIP) from 2015 to 2018 to describe the differences in 30-day complication incidence between smokers and non-smokers in two separate bariatric subpopulations—patients who underwent LSG and LRYGB.

## Material and methods

### Study design

This study was a retrospective, bigdata analysis of patients who underwent a Laparoscopic Sleeve Gastrectomy and Roux-en Y Gastric Bypass. All surgeries were done between January 1, 2015, and December 31, 2018, at centers participating in the MBSAQIP. The quality improvement program prospectively collects data on many variables, including standardized demographics, preoperative comorbidities, laboratory values, and 30-day postoperative mortality and morbidity outcomes for patients undergoing bariatric treatment in participating hospitals in the United States and Canada [4]. For this kind of research activity, IRB approval or written consent was not required.

### Study population—strategy selection

**Fig. 1:**
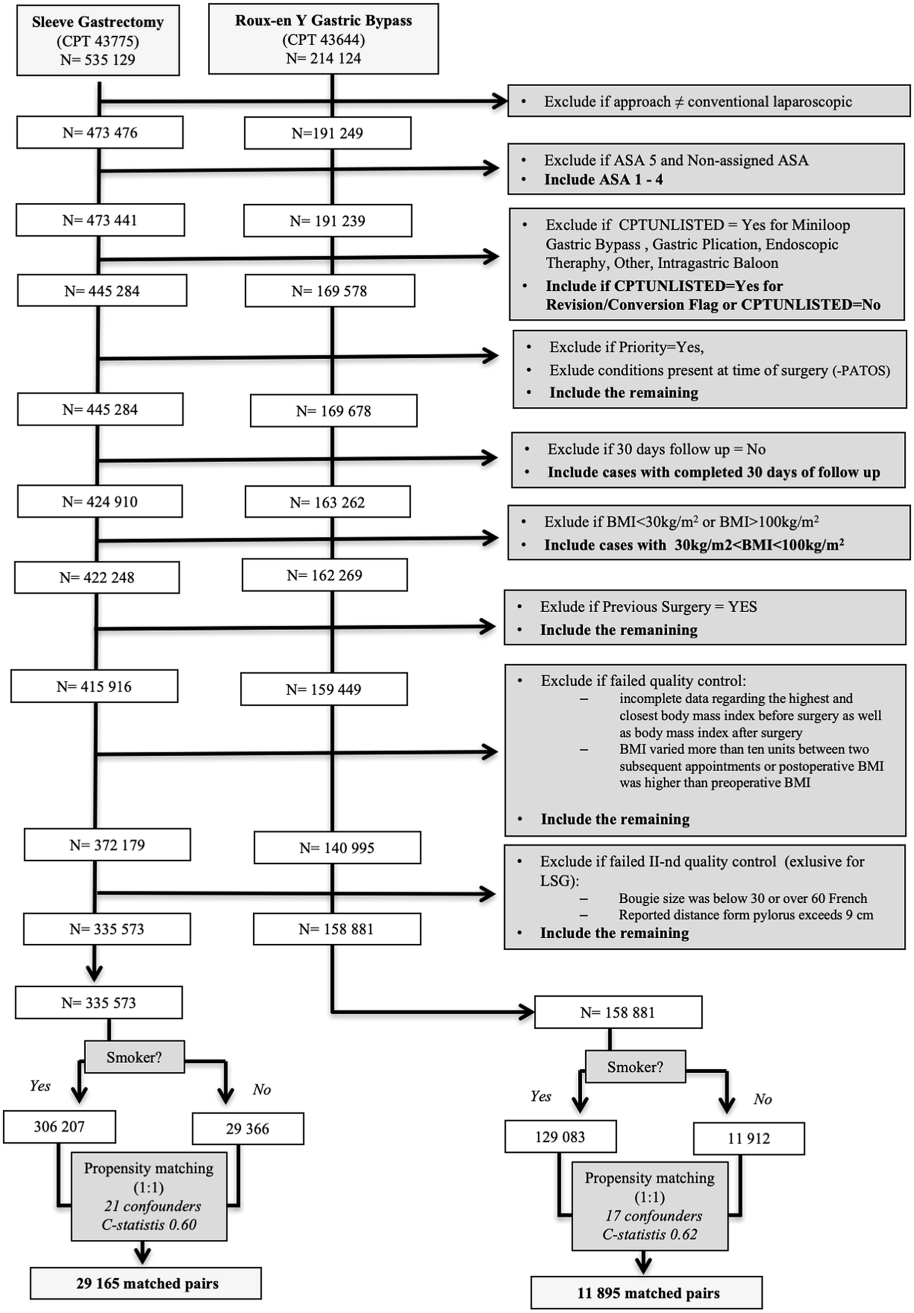
Flow chart of the study

The Strategy for Case Selection is presented in Fig. 1. MBSAQIP Participant User File (PUF) was utilized for the study. PUF file is a Health Insurance Portability and Accountability Act (HIPAA)-compliant data file containing cases submitted to the Metabolic and Bariatric Surgery Accreditation and Quality Improvement Program. The PUF contains patient-level, aggregate data and does not identify hospitals, health care providers, or patients. The definitions of variables are available in the official User Guide for the MBSAQIP [4]. According to this, the smoker was defined as *Current Smoker within one year*. The documents mentioned above are available on https://www.facs.org/quality-programs/mbsaqip/participant-use.

Data analysis was done using recommendations included in the *“Practical Guide to Surgical Data Sets: Metabolic and Bariatric Surgery Accreditation and Quality Program (MBSAQIP)"* published by Telem and Dimick [5]. *STROBE checklist* and *Checklist to Elevate the Science of Surgical Database Research* published by Haider et al. were applied [6, 7]. First, we identify all cases of patients who underwent sleeve gastrectomy or Roux-en-Y gastric bypass (as indicated by the use of 43,775 or 43,644 as the principal current procedural terminology [CPT] code for surgery type, according to the MBSAQIP PUF manual). In the next steps, we excluded cases whose initial surgical approach was listed as other than laparoscopic conventional. Other exclusions were as follows: American Society of Anesthesiologists (ASA) class of 5 or missing, cases with additional unlisted CPT codes (for Miniloop Gastric Bypass, Gastric Plication, Endoscopic Therapy, Other, Intragastric Baloon), revisional cases, emergency cases, conditions present at the time of surgery, lack of 30-day follow-up, and BMI below 30 or over 90 kg/m^2^.

Validation & quality tests were done on the cohorts to remove cases with the following criteria from the PUF 2015, PUF 2016, PUF 2017, and PUF 2018 datasets:

- observation with *incomplete data* regarding the highest and closest BMI before or after surgery;
- *BMI variation of more than 10 kg/m*^*2*^ between two subsequent postoperative appointments;
- *postoperative BMI > preoperative BMI;*
- for LSG cohort— reported *bougie size below 30 or over 60 French*. Bougie size range described in the literature is between 30 and 60 French.[8],
- for LSG cohort—reported *distance from pylorus exceeding 9 cm*. Distance > 10 cm was not described in the literature [9].

Finally, two cohorts were divided into groups regarding the smoking status within the last 12 months before surgery on smokers and non-smokers. Smokers cases were matched (1:1) with non-smokers controls in each cohort (separate for LSG and LRYGB patients) using propensity scoring to manage for potential confounding. Records with missing data were excluded from the analysis.

### Outcomes

#### All-cause mortality was the primary outcome

We also assessed the following secondary outcomes: operative time (OR), length of hospital stay (LoS), emergency department (ED) visits within 30 days postoperative, 30-day readmission, 30-day intervention, 30-day reoperation, leak rate (defined as drain present > 30 days, organ space surgical site infection, leak-related 30-day readmission, or leak-related 30-day reoperation or intervention), *bleeding event* (defined as bleed-related 30-day readmission, bleed-related 30-day reoperation, or transfusion required within 72 h postoperatively), and *30-day morbidity* (including unplanned admission rate to Intensive Care Unit within 30 days, pulmonary embolism, space surgical site infections, progressive renal insufficiency, postoperative sepsis, unplanned intubation, postoperative urinary tract infections, vein thrombosis requiring therapy*,* acute renal failure, postoperative cardiac arrest requiring CPR, coma over 24 h, stroke or cerebrovascular accident, postoperative deep incisional surgical site infections, postoperative myocardial infarction, postoperative ventilation, intraoperative nerve injury, pneumonia, postoperative septic shock, unplanned intubation, *Clostridium difficile* infection, and wound disruption). In the literature, there are pieces of evidence of increased vein thrombosis formation and postoperative pneumonia in smokers versus non-smokers [10, 11]. To re-assess the observations, we decided to investigate vein thrombosis requiring therapy, pulmonary embolism, and pneumonia as separate outcomes.

### Statistical analysis

In order to minimize selection bias, we used propensity scoring. We decided to use propensity scoring in the analysis because our primary outcome—mortality—was a rare event. Acknowledging multiple confounders, this approach was appropriate. The matching was based on the probability of being smokers within the last 12 months before surgery. Cases were matched with controls by 21 variables in the LSG cohort and by 17 variables in the LRYGB cohort. The matching was performed using a 1:1 greedy-matching algorithm, with a caliper of 0.05 standard deviation of the logit of the propensity score [12]. Standardized differences for all the baseline covariates after matching were assessed to check the postmatch balance. Standardized differences < 0.1 for a given variable indicate a small imbalance. In the matched cohorts, continuous outcomes were analyzed using the paired t-test or Wilcoxon signed ranks test. Dichotomous outcomes analyzed using McNemar’s analysis or Cochran–Mantel–Haenszel test. A description of effect estimates (risk ratio, RR) and 95% confidence interval (95% CI) was reported for category outcomes. The mean difference (or median) with 95% CI was reported for continuous variables. The analysis was done using SAS® software, University Edition (SAS 9.4 Institute Inc., Cary, NC, USA).

## Results

A total of Eighty-two thousand one hundred and twenty patients were included in the final analysis. Tables 1 and 2 present the baseline characteristics of analyzed patients in the both cohorts.

**Table 1:** Demographic characteristics for LSG subgroup

Characteristic	Original cohort			Matched cohort			
	Smokers *N* = 29 366	Non-smokers *N* = 306 207	*p *value	Smokers *N* = 29 165	Non-smokers *N* = 29 165	*p *value	Standardized differences
	Mean (SD) or % / Median Q1 and Q3			Mean (SD) or % / Median Q1 and Q3			
Age (years)	44.5 ± 12.1	41.7 ± 10.8	< 0.001	41.7 ± 10.8	41.5 ± 11.6	0.003	0.015
BMI (kg/m^2^)	46.0 ± 7.5	45.7 ± 7.6	< 0.001	46.0 ± 7.60	45.7 ± 7.8	0.340	0.002
Sex (female)	79.0%	79.5%	0.028	79.0%	78.8%	0.476	0.006
Race (white)	74.0%	71.7%	< 0.001	74.0%	73.9%	0.767	0.002
Pre-op hypertension	42.7%	47.1%	< 0.001	47.2%	42.4%	0.474	0.006
Pre-op diabetes mellitus	22.1%	23.2%	< 0.001	22.2%	22.2%	0.944	0.001
Pre-op hyperlipidemia	22.2%	22.5%	0.305	22.2%	22.1%	0.637	0.004
Pre-op obstructive sleep apnea*	37.6%	37.3%	0.351	37.7%	37.7%	1.000	0.007
GERD	29.6%	27.9%	< 0.001	29.5%	29.1%	0.268	0.009
PTC	1.8%	1.9%	0.573	1.8%	2.0%	0.249	0.010
Pre-op vein thrombosis requiring therapy	1.6%	1.6%	0.289	1.5%	1.5%	0.661	0.004
History of pulmonary embolism	1.1%	1.2%	0.611	1.1%	1.2%	0.938	0.001
Preoperative renal insufficiency	0.4%	0.6%	< 0.001	0.4%	0.4%	0.950	0.001
History of severe chronic obstructive pulmonary disease (COPD)	3.5%	1.4%	< 0.001	3.5%	3.5%	0.375	0.004
Steroid/immunosuppressant use for a chronic condition	1.9%	1.9%	0.929	1.8%	1.9%	0.361	0.008
Bariatric surgeon	76.3%	77.75	< 0.001	76.4%	76.2%	0.681	0.003
Sleeve oversew	21.7%	21.5%	0.466	21.3%	21.0%	0.436	0.007
Sleeve staple line reinforcement	67.3%	66.8%	0.144	67.1%	67.2%	0.825	0.002
Sleeve bougie size (Fr)	37.09 ± 2.92	37.09 ± 2.82	0.166	37.1 ± 2.8	37.0 ± 2.8	0.070	0.016
Sleeve distance to pylorus (cm)	4.89 ± 1.19	4.89 ± 1.21	0.245	4.9 ± 1.2	4.9 ± 1.2	0.798	0.001
ASA class			< 0.001				0.002
I	0.3	0.3		0.3%	0.2%		
II	21.8	24.4		21.8%	21.8%		
III	74.4	72.2		74.4%	74.4%		
IV	3.6	3.1		3.6%	3.6%		

*PCI* percutaneous coronary intervention, *PTCA* percutaneous transluminal coronary angioplasty

^*^No discordant pairs, *p *value was not calculated

**Table 2:** Outcomes for LSG subgroup

Outcomes	Smokers *N* = 29 165		Non-smokers *N* = 29 165		Mean difference ± SD	*p *value
Continuous	Mean ± SD		Mean ± SD			
Length of bariatric procedure (min)	69.9 ± 36.0		69.8 ± 33.2		−0.01 ± 1.78	0.438
Length of hospital stay (days)	1.5 ± 1.1		1.6 ± 1.4		0.04 ± 48.63	0.313

Categorical	%	*N*	%	*N*	Risk Ratio [95%CI]	P -value
Death within 30 days	0.02	6	0.04	13	RR: 0.46 [0.17–1.21]	0.108
Conversions to open approach	0.05%	16	0.07%	19	RR: 0.84 [0.43–1.64]	0.612
Emergency department (ED) visits within the 30 days postoperative*	7.70%	1365	6.58%	1167	RR: 1.17 [1.09–1.27]	< 0.001
Inpatient readmission(s) by midnight of POD 30	3.67%	1070	3.10%	905	RR: 1.18 [1.08–1.29]	< 0.001
Interventions performed within 30 days	1.03%	300	0.84%	246	RR: 1.22 [1.00–1.24]	0.020
Reoperations performed within 30 days	0.99%	289	0.79%	231	RR: 1.25 [1.05–1.48]	0.010
Leak within 30 days	0.59%	171	0.32%	93	RR: 1.83 [1.43–2.37]	< 0.001
Bleeding within 30 days	0.73%	213	0.70%	203	RR: 1.05 [0.87–1.27]	0.622
Any medical complications within 30 days	1.83%	535	1.63%	475	RR: 1.12 [0.99–1.27]	0.065
Pulmonary embolism	0.06%	18	0.08%	22	RR: 0.82 [0.44–1.52]	0.572
Vein thrombosis	0.21%	62	0.20	58	RR: 1.07 [0.75–1.53]	0.715
Pneumonia	0.23%	66	0.15%	45	RR: 1.15 [1.00–2.14]	0.046

^*^for 17 737 pairs

### Laparoscopic sleeve gastrectomy subgroup analysis

Smokers who underwent LSG were older (44.5 ± 12.1 vs. 41.7 ± 10.8 years, *p* < 0.001) and have higher prevalence of COPD (3.5% vs. 1.4%, *p* < 0.001), and GERD ( 29.6% vs. 27.9%, *p* < 0.001). However, BMI (44.6 ± 7.5 vs 45.7 ± 7.6, *p* < 0.001) and the prevalence of hypertension (42.7% vs. 47.1%, *p* < 0.001) and diabetes ( 22.1% vs. 23.2%, *p* < 0.001) was lower in the group when compared to non-smokers. After matching, 29 165 pairs were included in the analysis. The C-statistic for the model was 0.60. The standardized differences were < 0.1 for all variables, indicating a lack of significant differences between the two groups. (Table 1) Results for LSG subpopulation are presented in Table 2. Mortality was low and comparable between smokers and non-smokers (0.02% vs. 0.04%; relative risk [RR], 0.46; 95% confidence interval [CI], 0.17–1.21, *p* = 0.108). Smoking increased risk for emergency department visits rate (7.70% vs. 6.58%; RR, 1.17; 95%CI 1.09–1.27, *p* < 0.001), inpatients readmission rate (3.67% vs. 3.10%; RR, 1.18; 95%CI 1.08–1.29, *p* < 0.001), intervention rate (1.03% vs. 0.84%; RR, 1.22; 95%CI 1.00–1.24, *p* = 0.020), reoperation rate (0.99% vs. 0.79%; RR, 1.25; 95%CI 1.05–1.48, *p* = 0.010), and leak rate (0.59% vs. 0.32%; RR, 1.83; 95%CI 1.43–2.37, *p* < 0.001). Operative time (MD -0.01 [± 1.78] minutes, *p* = 0.438), length of hospital stay (MD 0.04 [± 48.63] days, *p* = 0.313), the conversion rate (0.05% vs. 0.07%; RR, 0.84; 95%CI 0.43–1.64, *p* = 0.612), bleeding rate (0.73% vs. 0.70%; RR, 1.05; 95%CI 0.87–1.27, *p* = 0.662), and morbidity rate (1.83% vs. 1.63%; RR, 1.12; 95%CI 0.99–1.23, *p* = 0.065) were comparable between groups. Smoking increased the risk for postoperative pneumonia (0.23% vs. 0.15%; RR: 1.15 [1.00–2.14], *p* = 0.046) but did not affect the risk for vein thrombosis (0.21% vs. 0.20%; RR: 1.07 [0.75–1.53], *p* = 0.715) and pulmonary embolism (0.06% vs. 0.08%; RR: 0.82 [0.44–1.52], *p* = 0.572). Three of the most common reasons for re-intervention were as follows: nausea and vomiting (22.3%), anastomotic/staple line leak 17.7%, and other [not listed] (17.3%). Likewise, in the control group, the most common were: nausea and vomiting (23.6%), other [not listed] (22.8%), and anastomotic/staple line leak (14.6%).

### Laparoscopic Roux-en Y gastric bypass subgroup analysis

Smokers who underwent LRYGB were younger (41.7 ± 10.5 vs. 45.4 ± 11.9, *p* < 0.001) and more likely have COPD (4.4% vs. 1.6%, *p* < 0.001), and GERD (40.8% vs. 39.0% *p* < 0.001). Their BMI was higher (46.9 ± 7.8 vs. 45.9 ± 7.7, *p* < 0.001). Yet, the prevalence of hypertension (47.2% vs. 52.8%, *p* < 0.001), diabetes (32.4% vs. 35.4%, *p* < 0.001), hyperlipidemia (26.4% vs. 29.2%, *p* < 0.001) and sleep apnea (43.7% vs. 44.9%, *p* < 0.001) was lower in the smokers when compared to non-smokers. After matching, 11 895 pairs were included in the ultimate analysis. The C-statistic for the model was 0.62. The standardized differences were below 0.1 for all variables, showing a lack of significant differences between the two groups. (Table 3) Results for LRYGB subpopulation are presented in Table 4. Mortality was very low and comparable between smokers and non-smokers (0.01% vs. 0.01%; relative risk [RR], 1.00 95% confidence interval [CI], 006–15.99, *p* = 1.00). Cases had significantly longer length of hospital stay (MD 0.05 [± 2.06] days, *p* < 0.001). Smoking increased risk for emergency department visits rate (12.54% vs. 9.68%; RR, 1.29; 95%CI 1.17–1.43, *p* < 0.001), inpatients readmission rate (7.54% vs. 5.88%; RR, 1.28; 95%CI 1.16–1.41, *p* < 0.001), intervention rate (3.53% vs. 2.30%; RR, 1.54; 95%CI 1.32–1.80, *p* < 001), reoperation rate (3.17% vs. 1.86%; RR, 1.70; 95%CI 1.45–2.00, *p* < 0.001), leak rate (1.05% vs. 0.59%; RR, 1.78; 95%CI 1.33–2.39, *p* < 0.001), bleed rate (2.03% vs. 1.45%; RR, 1.39; 95%CI 1.15–1.69, *p* < 0.001), and morbidity (4.20% vs. 3.38%; RR, 1.24; 95%CI 1.09–1.41, *p* = 0.001). Operative time (MD 1.1 [± 72.1] minutes, *p* = 0.090) and the conversion rate (0.38% vs. 0.36%; RR, 1.05; 95%CI 0.69–1.59, *p* = 0.831) were comparable between groups. Smoking did not affect the risk for pneumonia (0.36% vs. 0.35%; RR 1.00 [0.65–1.53]; *p* = 1.000), vein thrombosis (0.23% vs. 0.17%; RR: 1.35 [0.76–2.41], *p* = 0.307), and pulmonary embolism (0.11% vs. 0.14%; RR: 0.76 [0.37–1.57], *p* = 0.465). The most common reasons for re-intervention were as follows: Nausea and Vomiting, Fluid, Electrolyte or Nutritional Depletion (21.4%), Strictures/Stomal Obstruction (19.8%), and other [not listed] (14.5%). In controls, the most common were as follows: Strictures/Stomal Obstruction (24.8%), Nausea and Vomiting, Fluid, Electrolyte or Nutritional Depletion (18.7%), and other [not listed] (12.8%).

**Table 3:** Demographic characteristics for LRYGB subgroup

Characteristic	Original cohort			Matched cohort			
	Smokers *N* = 11 912	Non-smokers *N* = 129 083	*P *value	Smokers *N* = 11 895	Non-smokers *N* = 11 895	*P *value	Standardized differences
	Mean (SD) or % / Median Q1 and Q3			Mean (SD) or % / Median Q1 and Q3			
Age (years)	41.7 ± 10.5	45.4 ± 11.9	< 0.001	41.7 ± 10.5	41.8 ± 11.3	0.870	0.001
BMI (kg/m^2^)	46.9 ± 7.8	45.9 ± 7.7	< 0.001	46.9 ± 7.8	46.9 ± 8.1	0.195	0.003
Sex (female)	82.1%	80.2%	< 0.001	83.1%	82.4%	0.526	0.008
Race (white)	79.8%	76.3%	< 0.001	79.8%	90.0%	0.056	0.004
Pre-op hypertension	47.2%	52.8%	< 0.001	47.2%	47.6%	0.476	0.009
Pre-op diabetes mellitus	32.4%	35.4%	< 0.001	32.4%	32.5%	0.066	0.000
pre-op hyperlipidemia	26.4%	29.2%	< 0.001	26.4%	26.7%	0.598	0.007
Pre-op obstructive sleep apnea*	43.7%	44.9%	0.015	43.7%	43.7%	1.000	0.003
GERD	40.8%	39.0%	< 0.001	40.8%	40.8%	0.936	0.001
PTC	2.0%	2.3%	0.047	2.0%	1.9%	0.573	0.008
Pre-op vein thrombosis requiring therapy	2.2%	2.0%	0.195	2.2%	2.1%	0.687	0.006
History of pulmonary embolism	1.3%	1.3%	0.739	1.3%	1.3%	1.000	0.001
Preoperative renal insufficiency	0.5%	0.6%	0.015	0.4%	0.5%	0.624	0.008
History of severe chronic obstructive pulmonary disease (COPD)	4.4%	1.6%	< 0.001	4.4%	4.4%	0.951	0.001
Steroid/immunosuppressant use for a chronic condition	1.6%	1.6%	0.849	1.4%	1.6%	0.192	0.017
Bariatric surgeon	70.9%	71.9%	0.018	72.11	70.9%	0.044	0.026
ASA			0.010			0.286	0.010
I	0.2%	0.2%		0.2%	0.2%		
II	15.9%	15.3%		15.9%	15.6%		
III	80.0%	72.1%		80.0%	80.1%		
IV	3.9%	4.0%		3.9%	4.1%		

*PCI* Percutaneous coronary interventions, *PTCA* Percutaneous transluminal coronary angioplasty

^*^No discordant pairs, *p* value was not calculated

**Table 4:** Outcomes for LRYGB subgroup

Outcomes	Smokers *N* = 11 895		Non- Smokers *N* = 11 895		Mean difference ± SD	*p* value
Continuous	Mean ± SD		Mean ± SD			
Length of bariatric procedure (min)	117.7 ± 51.9		116.6 ± 51.3		1.1 ± 72.1	0.090
Length of hospital stay (days)	2.0 ± 1.4		1.9 ± 1.5		0.05 ± 2.06	< 0.001

Categorical	%	*N*	%	*N*	Risk Ratio [95%CI]	*p* value
Death within 30 days	0.01%	1	0.01%	1	RR: 1.00 [0.06–15.99]	1.000
Conversions to open approach	0.38%	45	0.36%	43	RR: 1.05 [0.69–1.59]	0.831
Emergency department (ED) visits within the 30 days postoperative *	12.54%	813	9.68%	628	RR: 1.29 [1.17–1.43]	< 0.001
Inpatient readmission(s) by midnight of POD 30	7.54%	897	5.88%	700	RR: 1.28 [1.16–1.41]	< 0.001
Interventions performed within 30 days	3.53%	420	2.30%	274	RR: 1.54 [1.32–1.80]	< 0.001
Reoperations performed within 30 days	3.17%	377	1.86%	221	RR: 1.70 [1.45–2.00]	< 0.001
Leak within 30 days	1.05%	125	0.59%	70	RR: 1.78 [1.33–2.39]	< 0.001
Bleeding within 30 days	2.03%	241	1.45%	173	RR: 1.39 [1.15–1.69]	< 0.001
Any medical complications within 30 days	4.20%	499	3.38%	402	RR: 1.24 [1.09–1.41]	0.001
Pulmonary embolism	0.11%	13	0.14%	17	RR: 0.76 [0.37–1.57]	0.465
Vein thrombosis	0.23%	27	0.17%	20	RR: 1.35 [0.76–2.41]	0.307
Pneumonia	0.36%	43	0.35%	43	RR: 1.00 [0.65–1.53]	1.000

^*^for 6485 pairs

## Discussion

Our analysis revealed that a history of smoking within 12 months before surgery increased the risk significantly for surgical complications in bariatric patients who underwent laparoscopic sleeve gastrectomy or laparoscopic Roux-Y gastric bypass. However, it did not have any effect on mortality. We estimated the effect size on several essential complications. We are pointing out that exposure to tobacco affects the most the risk for leakage. Smokers who had the LSG had an 84% increase in the risk of getting a postoperative leak. Similarly, smokers after LRYGB had a 78% increase in leak risk over the unexposed group. What is more interesting, we observed in the LRYGB subgroup that smoking increased risk for bleeding and morbidity. The relationships were not present in the LSG cohort.

The findings contribute to the literature by examining the effects of tobacco use as they relate to solely LSG and LRYGB. Moreover, our study utilized the most significant bariatric registry—there is no other study on such an enormous population. The lower incidence of diabetes and hypertension in smokers before matching seems counterintuitive. Unfortunately, we cannot find a reasonable explanation of this observation. The thought-provoking is that increased risk for surgical complications did not affect the mortality in the analysis. A possible explanation is that pulmonary embolism is the most prevalent cause of death in the early perioperative period [13]. In our analysis, this outcome was comparable between smokers and non-smokers. Perhaps the life-threatening consequences of increased leakage could be captured beyond 30 days of observation and affect mortality. Future studies should address this issue.

Smokers are very often excluded from consideration for bariatric surgery. In analyzed population, only 8.45% gastric bypass patients and 8.75% sleeve patients reported smoking within last 12 months before surgery. However, as the study by Laura J. Rasmussen-Torvik et al. proved, this group may still benefit from the bariatric treatment [14]. A retrospective cohort study based on large Israeli integrated health care provider registry showed that bariatric surgery was associated with significantly lower mortality in both smokers and non-smokers. As the authors stated, if this conclusion is replicated in other studies, it will change the policy regarding current smokers.

So far, smoking is considered to be a risk factor for surgical complications. Haskin et al. investigated the effect of smoking on bariatric surgical outcomes using the National Surgical Quality Improvement Program (NSQIP) between 2005 and 2010. Using different methodology, authors reached the conclusion that in the laparoscopic surgery subgroup, smokers had a significantly increased incidence of prolonged intubation, reintubation, sepsis, shock, and length of stay. In their analysis, smoking did not significantly increase the risk of mortality for patients undergoing bariatric surgery [3].

Aminian et al., in the broad analysis of risk factors for bariatric patients with diabetes type 2 based on MBSAQIP, concluded that smoking should be considered as a modifiable risk factor for early complications after bariatric surgery [15].

The timing of abstinence from smoking is under the debate right now. Wong et al. suggested that at least four-week abstinence should be recommended to reduce the risk of wound healing and postoperative respiratory complications [1]. Inadomi et al. based on the analysis of Michigan Bariatric Surgery Collaborative data proposed the minimum required length of preoperative smoking cessation should be no longer than one year for RYGB and three months for SG [16]. Our analysis showed smoking within the 12 months before surgery affected the risk for surgical complications significantly. Based on that, we stated that at least one-year abstinence should be recommended. It may be difficult in clinical practice, but it is necessary to reduce the risk of postoperative complications, especially the leaks.

Our study has a several limitations. First, the MBSAQIP database is observational. The relationship between adverse events should be tested prospectively in a controlled environment to evaluate a potential causal association. However, considering the low rate of adverse events, it would be nearly impossible to conduct a randomized control study with enough power to show a difference. Second, smoking status was self-reported by patients. The study by Wolvers et al. determined the accuracy of self-reported smoking compared to cotinine measurement in three phases of the bariatric surgery trajectory showed that underreporting of smoking occurs before bariatric surgery, mainly on the day of surgery [17]. Future studies should implement objective measurement to assess the smoking cessation. Third, they were not able to assess the efficacy bariatric treatment between smokers and non-smokers because of the lack of data on weight loss outcomes and comorbidities improvement. Yet, Kowalewski et al. proved that smoking status was not significantly associated with weight loss in long-term observation in sleeve patients [18].

## Conclusion

Our study shows that smoking within the last 12 months before surgery increased the risk for postoperative complications in patients who underwent laparoscopic Roux-Y gastric bypass and laparoscopic sleeve gastrectomy. The increase in risk was the most prevalent in the case of staple line leak or anastomotic leakage. However, it did not affect all-cause mortality.
